# Adrenocortical Challenge Response and Genomic Analyses in Scottish Terriers With Increased Alkaline Phosphate Activity

**DOI:** 10.3389/fvets.2018.00231

**Published:** 2018-10-09

**Authors:** Kurt L. Zimmerman, David L. Panciera, Ina Hoeschele, W. Edward Monroe, Stephanie Michelle Todd, Stephen R. Werre, Tanya LeRoith, Kellie Fecteau, Bathilda B. Lake

**Affiliations:** ^1^Department of Biomedical Sciences and Pathobiology, Virginia Maryland College of Veterinary Medicine, Virginia Tech, Blacksburg, VA, United States; ^2^Department of Small Animal Clinical Sciences, Virginia Maryland College of Veterinary Medicine, Virginia Tech, Blacksburg, VA, United States; ^3^Department of Statistics, College of Science, and Biocomplexity Institute, Virginia Tech, Blacksburg, VA, United States; ^4^Veterinary Medicine Experiment Station, Virginia Maryland College of Veterinary Medicine, Virginia Tech, Blacksburg, VA, United States; ^5^Study Design and Statistical Analysis Laboratory, Virginia Maryland College of Veterinary Medicine, Virginia Tech, Blacksburg, VA, United States; ^6^Department of Biomedical and Diagnostic Sciences, College of Veterinary Medicine, University of Tennessee, Knoxville, TN, United States

**Keywords:** hyperadrenocorticism, single nucleotide polymorphism, steroidogenesis, 17β-hydroxysteroid dehydrogenase 2, ALP

## Abstract

Scottish terriers (ST) frequently have increased serum alkaline phosphatase (ALP) of the steroid isoform. Many of these also have high serum concentrations of adrenal sex steroids. The study's objective was to determine the cause of increased sex steroids in ST with increased ALP. Adrenal gland suppression and stimulation were compared by low dose dexamethasone (LDDS), human chorionic gonadotropin (HCG) and adrenocorticotropic hormone (ACTH) response tests. Resting plasma pituitary hormones were measured. Steroidogenesis-related mRNA expression was evaluated in six ST with increased ALP, eight dogs of other breeds with pituitary-dependent hyperadrenocorticism (HAC), and seven normal dogs. The genome-wide association of single nucleotide polymorphisms (SNP) with ALP activity was evaluated in 168 ST. ALP (reference interval 8–70 U/L) was high in all ST (1,054 U/L) and HAC (985 U/L) dogs. All HAC dogs and 2/8 ST had increased cortisol post-ACTH administration. All ST and 2/7 Normal dogs had increased sex steroids post-ACTH. ST and Normal dogs had similar post-challenge adrenal steroid profiles following LDDS and HCG. Surprisingly, mRNA of hydroxysteroid 17-beta dehydrogenase 2 (HSD17B2) was lower in ST and Normal dogs than HAC. HSD17B2 facilities metabolism of sex steroids. A SNP region was identified on chromosome 5 in proximity to HSD17B2 that correlated with increased serum ALP. ST in this study with increased ALP had a normal pituitary-adrenal axis in relationship to glucocorticoids and luteinizing hormone. We speculate the identified SNP and HSD17B2 gene may have a role in the pathogenesis of elevated sex steroids and ALP in ST.

## Introduction

Scottish terriers (ST) have a high prevalence of elevated serum alkaline phosphatase (ALP) activity (hyperphosphatasemia) (1, 2). The corticosteroid isoform of alkaline phosphatase predominates in these dogs which, along with low urine specific gravity and the hepatocellular cytoplasmic reticulation and vacuolation present on histopathology, is consistent with excessive corticosteroids (3). Evaluation of adrenocortical function in a prospective study confirmed steroid hormone excess in all 17 asymptomatic ST with hyperphosphatasemia studied. Hormone concentrations of cortisol, progesterone, 17-hydroxyprogesterone, and androstenedione after ACTH administration were elevated relative to sex-indexed reference intervals in 6, 12, 12, and 3 dogs, respectively (3). All dogs tested had one or more of these hormones elevated. Another study, although performed retrospectively, documented elevated post-ACTH cortisol or sex hormone concentrations in 68 and 88% of ST, respectively, tested because of clinical evidence suggestive of hyperadrenocorticism (2).

Some dogs with hyperadrenocorticism (HAC) have normal cortisol concentrations after ACTH administration, but have elevation of non-cortisol hormones (4). The pathogenesis of this phenomenon is not clear, but it has been proposed that a deficiency in some steroidogenic enzymes in hyperplastic or neoplastic adrenal glands could be present. This has led to evaluation of basal and post-ACTH serum concentrations of progesterone, 17-hydroxyprogesterone, androstenedione, estradiol, and testosterone in dogs suspected of having hyperadrenocorticism but without demonstrable abnormalities in cortisol secretion on standard tests (5–7). The sensitivity and specificity of post-ACTH concentrations of cortisol, progesterone, and 17-hydroxyprogesterone were found to be similar in dogs suspected of hyperadrenocorticism (4).

While excessive steroid hormone secretion appears to be the cause of the elevated ALP and vacuolar hepatopathy, the underlying mechanism resulting in hormone excess is unknown in ST. Hyperadrenocorticism can be due to an ACTH secreting pituitary tumor (pituitary- or ACTH-dependent) or can result from adrenal gland abnormalities (ACTH-independent) (8–10). Most cases of canine hyperadrenocorticism are pituitary-dependent, while 15–20% result from functional adrenocortical tumors (11). In humans, ACTH-independent adrenal hyperplasia is another, albeit uncommon, cause of hyperadrenocorticism (12–17). This form of glucocorticoid excess results from expression of illicit receptors by adrenocortical cells. These receptors include those to luteinizing hormone (LH), gastric inhibitory peptide, vasopressin, catecholamines, and serotonin. Food responsive hyperadrenocorticism, purportedly resulting from an abnormal adrenocortical response to gastric inhibitory peptide, has been reported in a dog with ACTH independent hyperadrenocorticism (18–20). Differentiating these forms of hyperadrenocorticism requires systematic testing.

Another condition that results in steroid hormone excess, congenital adrenal hyperplasia (CAH) comprises a group of inherited disorders of adrenal steroidogenesis in humans (21–24). While most cases of CAH are the result of an autosomal recessive deficiency of 21-hydroxylase, inherited abnormalities of other enzymes, including 17α-hydroxylase, 11β-hydroxylase, and 3β-hydroxysteroid dehydrogenase, also occur. These adrenal enzyme deficiencies result in decreased cortisol secretion, thus stimulating ACTH secretion (25, 26). The increase in ACTH secretion results in adrenocortical hyperplasia and increased production of adrenal androgens. A similar pathogenesis was hypothesized in Pomeranian dogs, but no enzyme mutation was identified, and other studies have not supported this (27, 28).

The purpose of the present study was to explore the origin of the abnormal steroid hormone secretion in ST with elevated ALP. In the first part of this study, we tested the hypothesis that excessive secretion of steroid hormones was the result of an ACTH-independent abnormal adrenocortical response to luteinizing hormone. The hypothalamic-pituitary-adrenal axis was interrogated by evaluating plasma ACTH concentration and by measuring the response of cortisol and non-cortisol adrenocortical hormones to administration of a low dose of dexamethasone in ST with elevated serum ALP activity, and other breeds of dogs with and without pituitary-dependent hyperadrenocorticism. To evaluate the role of LH in the abnormal adrenocortical function of ST, serum LH concentration and the adrenocortical hormone response to administration of human chorionic gonadotropin (HCG) were evaluated.

In the second part of this work, we performed two exploratory genome-wide analyses: mRNA expression profiling and a Genome-Wide Association Study (GWAS) of Single Nucleotide Polymorphisms (SNPs) of ST with elevated serum ALP activity in an attempt to better understand its pathogenesis. The goal of a GWAS is to identify genes that affect the risk of developing a disorder (in this case increased ALP and HAC). This is accomplished by utilizing SNPs (two different alleles occurring in a population at many positions in the genome). Dog breeds represent isolated populations with much more extensive linkage disequilibrium (correlation among genetic polymorphsisms) compared to humans, which allows conducting a GWAS with much smaller sample sizes (typically between 100 and 250 samples).

## Materials and methods

This study was approved by the Institutional Animal Care and Use Committee of Virginia Tech. Dogs included in this study were client owned and enrolled with signed consent. Participants were recruited from the Virginia-Maryland College of Veterinary Medicine, Veterinary Teaching Hospital (VTH) cliental. The study was conducted from 2013 to 2015. Raw data supporting the conclusions of this manuscript were made available at the Virginia Tech, VTechData data repository, DOI: 10.7294/5mx0-qs21, https://doi.org/10.7294/5mx0-qs21.

### Part 1: routine and endocrine

#### Animals

Three groups of dogs were included in part 1: ST with elevated serum ALP—group ST, dogs of other breeds with pituitary-dependent hyperadrenocorticism—group HAC, and healthy dogs of other breeds that acted as controls—group Normal. Dogs in the ST group had no clinically significant historical or physical examination abnormalities, serum ALP activity >210 U/L (arbitrarily set at 3 times the upper limit of the reference interval of 8–70 U/L), normal serum bilirubin concentration (3.42–6.84 umol/L) and no history of polydipsia (PD) or polyuria (PU). HAC dogs had clinical findings consistent with the diagnosis: PU/PD, polyphagia, hepatomegaly, obesity, alopecia, and muscle wasting, elevated serum ALP activity, failure of serum cortisol concentration to suppress normally on a low dose dexamethasone suppression test, and bilateral symmetrical adrenal enlargement on ultrasound. Dogs in the control group had no significant abnormalities on history or physical examination, CBC, serum biochemistries, urinalysis, ultrasound or cortisol response following dexamethasone suppression. Dogs receiving topical or systemic glucocorticoids, or anticonvulsants in the previous 90 days were excluded from the study.

#### Clinical and hormonal testing

In all dogs, CBC, serum chemistry, fasting bile acids, urinalysis, plasma adrenocorticotrophic hormone (ACTH), urine cortisol:creatinine ratio (UCCR), and abdominal ultrasound were evaluated. Urine samples for measurement of the UCCR were obtained by dog owners at home prior to presentation to the VTH. Adrenocortical function was assessed by obtaining blood samples immediately before and 4 and 8 h after administration of dexamethasone sodium phosphate (0.01 mg/kg IV, Dexamethasone SP, Bimeda-MTC Animal Health, Cambridge, Ontario). After obtaining the 8-h blood sample, cosyntropin (5 ug/kg IM, Cortrosyn, Amphastar Pharmaceuticals, Inc., Rancho Cucamonga, CA) was administered and another blood sample was obtained 1 h later (29, 30). Serum adrenal steroid profiles consisting of cortisol, androstenedione, estradiol, progesterone, 17-OH progesterone, and aldosterone were measured in all samples.

Two weeks after the initial evaluation, all dogs underwent sample collection for measurement of adrenal steroid profiles before and 2 and 4 h after intramuscular administration of HCG (50 IU/kg, HCG–Chorulon, Intervet Inc., Summit, NJ). In addition, resting serum concentrations of luteinizing hormone (LH), prolactin (PL), growth hormone (GH), and thyroid stimulating hormone (TSH) were measured to determine if any of these pituitary hormones where associated with the abnormal steroid hormones or ALP in ST.

### Part 2: genomic

#### RNA

For mRNA sequencing, the same dogs/groups (ST, HAC, Normal) as described in Part 1 were used for this portion of the genomic study. Whole blood EDTA samples were collected from all study dogs at time of initial examination for buffy coat transcriptome analysis. The EDTA blood was centrifuged 10 min at 23°C at 300 × g followed by buffy coat pipet extraction. Guanidinium thiocyanate-phenol-chloroform (TRIzol, Invitrogen, Carlsbad, CA) was added to the buffy coat at the ratio of 750 μl per 250 μl buffy coat then mixed thoroughly by inversion until homogeneous. Samples were stored at −80°C until batch processed for mRNA extraction and sequencing.

#### SNP and GWAS

For the GWAS and SNP study, a new cohort of Scottish terriers were enrolled. These dogs were recruited from across the USA. Inclusion criteria were Scottish terrier dogs between 1 and 12 years of age of either sex and spay-neuter status. These dogs were identified as being healthy by their owners and as not having received glucocorticoid or anti-convulsant medications within 3 months prior to enrollment. Venous EDTA (5 ml) and serum separator (5 ml) blood samples were collected by referring veterinarians. Serum separator tubes were centrifuged after 30 min for 10 min. Both samples were refrigerated at 40°C then shipped on ice overnight to the author (Kurt Zimmerman). Total bilirubin concentration and ALP activity were measured on the serum sample. Cases with total bilirubin results above the established upper reference interval (0.2–0.4 mg/dL, Virginia Tech Animal Laboratory Services (ViTALS), Blacksburg, VA) were excluded. The EDTA whole blood sample was frozen at −80°C upon arrival then batched for DNA extraction and SNP microarray analysis.

### Laboratory and assay information

#### Routine

Complete blood count (Advia 120, Siemens Medical Solutions USA, Inc., Malvern, PA), serum chemistries (AU480, Beckman Coulter, Brea, CA), ACTH (Immulite 1000, Siemens Medical Solutions USA, Inc., Malvern, PA) and urinalysis were performed at ViTALS. Urine cortisol and creatinine assays were performed by Michigan State University Diagnostic Center for Population and Animal Health, Lansing, MI (31).

#### Endocrine

Adrenal steroid profiles were performed by the Clinical Endocrinology Service at the University of Tennessee, College of Veterinary Medicine, Knoxville, TN (32). Radioimmunoassays for LH, PL, GH and TSH were performed at the Animal and Food Sciences Laboratory, Louisiana State University Agricultural Center (LSU AgCenter), Baton Rouge, LA using raised rabbit antibodies and sheep anti-rabbit gamma globulin; canine LH, PL, GH, and TSH standards were provided by Harbor-UCLA Research and Education Institute, Torrance, CA and LSU AgCenter, Baton Rouge, LA (33). Hormone assays from ViTALS, Michigan, Tennessee, and LSU AgCenter were validated by the respective laboratories.

#### RNA

mRNA extraction and sequencing were done by the Genomics Sequencing Center of the Biocomplexity Institute at Virginia Tech, Blacksburg, VA, for the 21 dogs in Part 1. Blood globulin RNA was removed from the buffy coat, TRIzol mixture using commercial kits (Ribo-Zero rRNA removal kit and the Globin-Zero rRNA/globin mRNA removal kit, Epicenter/Illumina, San Diego, CA). Stranded RNA-Seq Library Construction: Library preparations were performed on an Apollo 324 Robot (Wafergen, CA). One microgram of total RNA was depleted of globin and ribosomal RNAs using Illumina's Globin-Zero Gold Kit (Illumina, P/N GZG 1224). PolyA RNA was then converted into a library of template molecules using PrepX RNA-Seq for Illumina Library Kit, 48 samples (P/N 400046, Wafergen, Fremont, CA) for subsequent cluster generation and sequencing by an Illumina HiSeq. Poly-A mRNA was fragmented into smaller pieces (~140 nt). 3′ and 5′ adapters were ligated to the cleaved RNA fragments and converted to first strand cDNA using reverse transcriptase, followed by second strand synthesis. The products were purified and enriched with 13 cycles of PCR to create the final cDNA library. The 280–300 bp libraries (160–180 bp inserts) generated were validated using an Agilent 2100 Bioanalyzer (Agilent Technologies, Santa Clara CA) and quantified using Quant-iT dsDNA HS Kit (Invitrogen) and qPCR. Six individually indexed cDNA libraries were pooled and sequenced on an Illumina HiSeq to obtain a minimum of 60 million paired end reads. Cluster Generation and HiSeq Sequencing: Libraries were clustered onto a flow cell using Illumina's TruSeq PE Cluster Kit v3-cBOT-HS (PE-401-3001) and sequenced 2 × 100 Paired End using TruSeq SBS Kit v3-HS (200-cycles) (FC-401-3001). Following sequencing, data was trimmed for both adaptor and quality using a combination of ea-utils and Btrim (34, 35). Sequencing reads were then aligned to the genome using TopHat2/Bowtie2 (36, 37).

#### SNP and GWAS

DNA samples for SNP genotyping were prepared and processed by the Center for Genomics & Personalized Medicine Research, Wake Forest School of Medicine, Winston-Salem, NC. DNA was isolated from whole blood using the AutoPure LS automated system (Qiagen, Inc., Valencia, CA). SNP genotyping was performed using the CanineHD Genotyping BeadChip (Illumina, Inc., San Diego, CA) designed to be used with a diverse set of breeds, which contains 173,662 SNPs. BeadChips were scanned with the HiScan scanner (Illumina, Inc., San Diego, CA). The dogs were randomized to 14 chips (12 dogs per chip) such that 11 chips had 7 cases and 5 controls, and 3 chips had 6 cases and 6 controls (95 cases: dogs with ALP >150; 73 controls: dogs with ALP < 150). The randomization was performed within age groups resulting in similar average age across the 14 chips (range 6.8–7.3 years). Quality control included removal of SNPs with no chromosome assignment, SNPs on the X chromosome, SNPs with >1 genotype missing, non-polymorphic SNPs, and SNPs with no heterozygotes.

### Statistical analysis

#### Routine and endocrine

For conventional laboratory analytes, commercial software was used for the statistical analysis (Minitab 15, Minitab Inc., State College, Pa; JMP & SAS version 9.4, SAS Inc., Cary, NC; GraphPad Prisim 5, GraphPad Software Inc., La Jolla, CA). Adrenal steroid hormones with gender specific reference intervals were normalized for statistical comparison by the following formula: normalized result = (result – [{lower reference interval + upper reference interval}/2])/([upper reference interval – lower reference interval value]/2) such that normalized reference intervals were −1 to 1 for relevant steroid hormones. Percent amount of hormones was determined by comparing measured values at specified time points with base line concentrations. Normality distributions were accessed by normal probability plots and log transformed when appropriate. Age, ALP and Pituitary analytes were compared between groups using one-way analysis of variance. Overall *p*-values for each analyte were adjusted for multiple testing using the Benjamini–Hochberg (BH) False Discovery Rate method at level 0.05 (38). For analytes with significant BH adjusted *p*-values, all two-way comparisons between groups were performed using Tukey's procedure. For adrenal analytes, effects of group and time were assessed using mixed-model analysis of variance. The linear model specified group, time, and the interaction between group and time as fixed effects while dog identification within group was specified as a random effect. Overall *p*-values were also adjusted for multiple testing using the BH False Discovery Rate method. Where appropriate all 2-way comparisons were performed using Tukey's procedure. Between- and within-group comparisons were performed by slicing the interaction term between group and time.

#### RNA

mRNA data pre-processing and analyses was performed in *R* (http://www.r-project.org/) using *Bioconductor* (http://www.bioconductor.org/) packages (39, 40). For the raw count matrix, a total of 18,221 distinct transcripts had a non-zero count. The total read counts per dog ranged from near 3 million to over 15 million (median value just under 8 million) with one dog removed due to a low total count. Counts were converted to Counts Per Million (CPM) using the *cpm* function of the *edgeR* package, and all transcripts with CPM ≥0.25 in ≥2 dogs were retained, resulting in a count matrix for 15,141 transcripts and 18 dogs (39). The count data were normalized using the trimmed mean of M-values (TMM) method (41). The 18 dogs represented the three groups in phase 1: Group Normal (*n* = 5), Group HAC (*n* = 6), and Group ST (*n* = 7). Differential expression analysis was performed for the three pairwise comparisons C vs. N (C vs. N), S vs. N (S vs. N), and S vs. C (S vs. C). Two methods known to perform well with small sample sizes were used: (1) Empirical Bayes weighted linear model analysis using the logged CPM values as the response variable using the *limma R* package (42, 43); (2) a generalized linear model analysis using the negative binomial distribution as implemented in the *edgeR* package (39). Each of the three comparisons (HAC vs. Normal, ST vs. Normal and ST vs. HAC) was performed on the 15,141 transcripts, and multiple testing adjustment was achieved using the BH and *q*-value methods (44).

#### SNP and GWAS

For the GWAS study, SNP genotype analyses were performed in *R* (http://www.r-project.org). Part of the quality control analysis and the GWAS analysis were performed using the *R* package *GenABEL* (http://www.genabel.org) (45–48). For each SNP, a statistical test was performed to determine whether one of its alleles likely increased the risk for having elevated serum ALP. Such statistical tests are biased in the presence of population structure (as measured by genomic inflation). Population structure occurs mainly when samples are taken from a population which consists of distinct sub-populations and/or when samples are taken from a genetically isolated population such as a dog breed, in which individuals can be related to each other to varying degrees. To account for population structure, in particular for potential relatedness of some dogs, mixed model based statistical tests for association between each SNP and the phenotype (disease) as recommended and implemented in GenABEL were computed (including GRAMMAS and GRAMMAR-gamma) (49, 50). Mixed model analysis incorporated the kinships (genetic relationships) among all dogs, which were estimated from the SNP data (this reduced genomic inflation from high to moderate). Included as covariates in the mixed model analyses were the linear effect of age or the linear and quadratic effects of age (gender was not significant). Because in GWAS statistical tests are performed at each SNP, there is a need to adjust for multiple testing to control the number of false positive results. This is accomplished by computing a genome-wide and a suggestive significance threshold and reporting only those SNPs which fall below either of the two thresholds as candidates for further investigation. A common approach to computing the genome-wide threshold is to divide the significance level of 0.05 by the number of Linkage Disequilibrium (LD) blocks, where an LD block is a subset of consecutive SNPs which are highly correlated (44). The number of LD blocks was estimated using the *R* package *trio* (47) and was equal to 13,540, yielding a *p*-value threshold of (0.05/13,540) = 3.692762e-06 (which is in the range of *p*-value thresholds computed in several recent dog GWAS studies). The LD blocks included singleton SNPs. The suggestive significance threshold was computed as (1/13,540) = 7.385524e-05. SNPs with *p*-values below the suggestive threshold are worth reporting but are more likely to be false positives than SNPs with *p*-values below the genome-wide threshold. The main response variable used for the GWAS was the case-control status of the dogs, and the (logarithm base 2 transformed) ALP values were used as a secondary response.

## Results

### Part 1: routine and endocrine

#### Signalment, clinical, and routine laboratory testing

**Figure 1:**
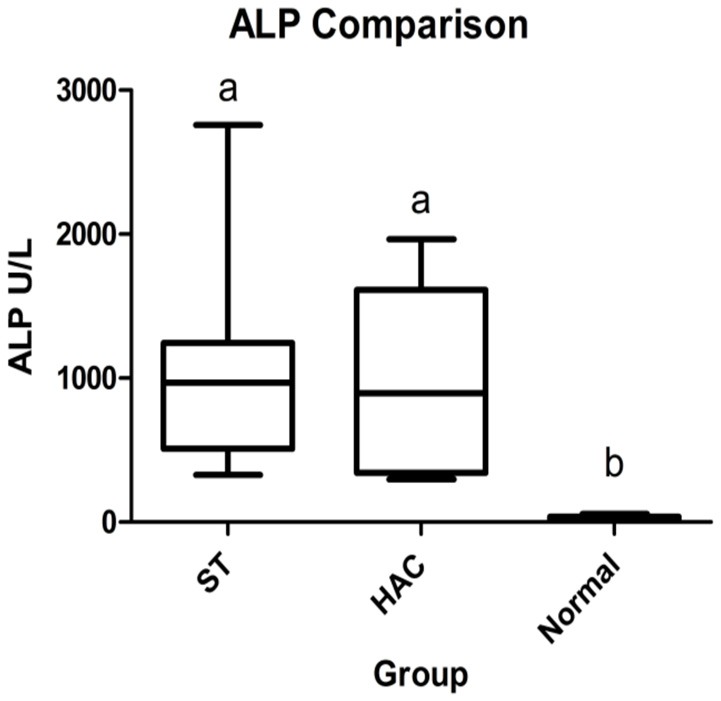
Box and whisker plots of serum alkaline phosphatase (ALP) data between dogs in the Scottish terrier (ST), hyperadrenocorticism (HAC), and control (Normal) groups; whiskers min to max, box Q_1_-Q_3_, bar represents median value, groups with differing lowercase letter superscripts are significantly different (*p* < 0.05).

The 21 dogs enrolled in the study comprised 8 ST with elevated ALP (3 neutered male and 5 spayed female) with a mean ± *SD* age of 10 ± 1.5 years, 6 dogs with HAC (3 neutered male and 3 spayed female) 10.2 ± 2.4 years of age, and 7 normal dogs (4 neutered male and 3 spayed female) 5.4 ± 2.3 years of age. Breeds in the HAC group included 2 mixed breed, and 1 each Miniature Schnauzer, Jack Russell Terrier, Basset Hound, Lhasa Apso. The normal group included 4 mixed breed and 3 Golden retrievers. There was no age difference between the ST and HAC groups; however, both these groups were older than the normal dogs. Dogs with HAC had clinical findings consistent with the diagnosis, including PU/PD (*n* = 5), polyphagia (*n* = 5), hepatomegaly (*n* = 5), obesity (*n* = 5), alopecia (*n* = 2), and muscle wasting (*n* = 2). The mean/median serum cortisol concentration 8 h after administration of dexamethasone was higher in the HAC group as compared to the ST and control dogs (HAC 58/43 ng/mL, ST 5.4/5.2 ng/mL, Normal 5.7/3.5 ng/mL, reference interval < 10 ng/mL). Bilateral adrenomegaly was found on ultrasound examination in all dogs in the HAC group, consistent with PDH. Mean/median ST and HAC serum ALP were higher than for control dogs (ST 1,054/966 U/L, HAC 985/897 U/L, Normal 26/20, reference interval 8–70 U/L), Figure 1.

#### Adrenal

**Figure 2:**
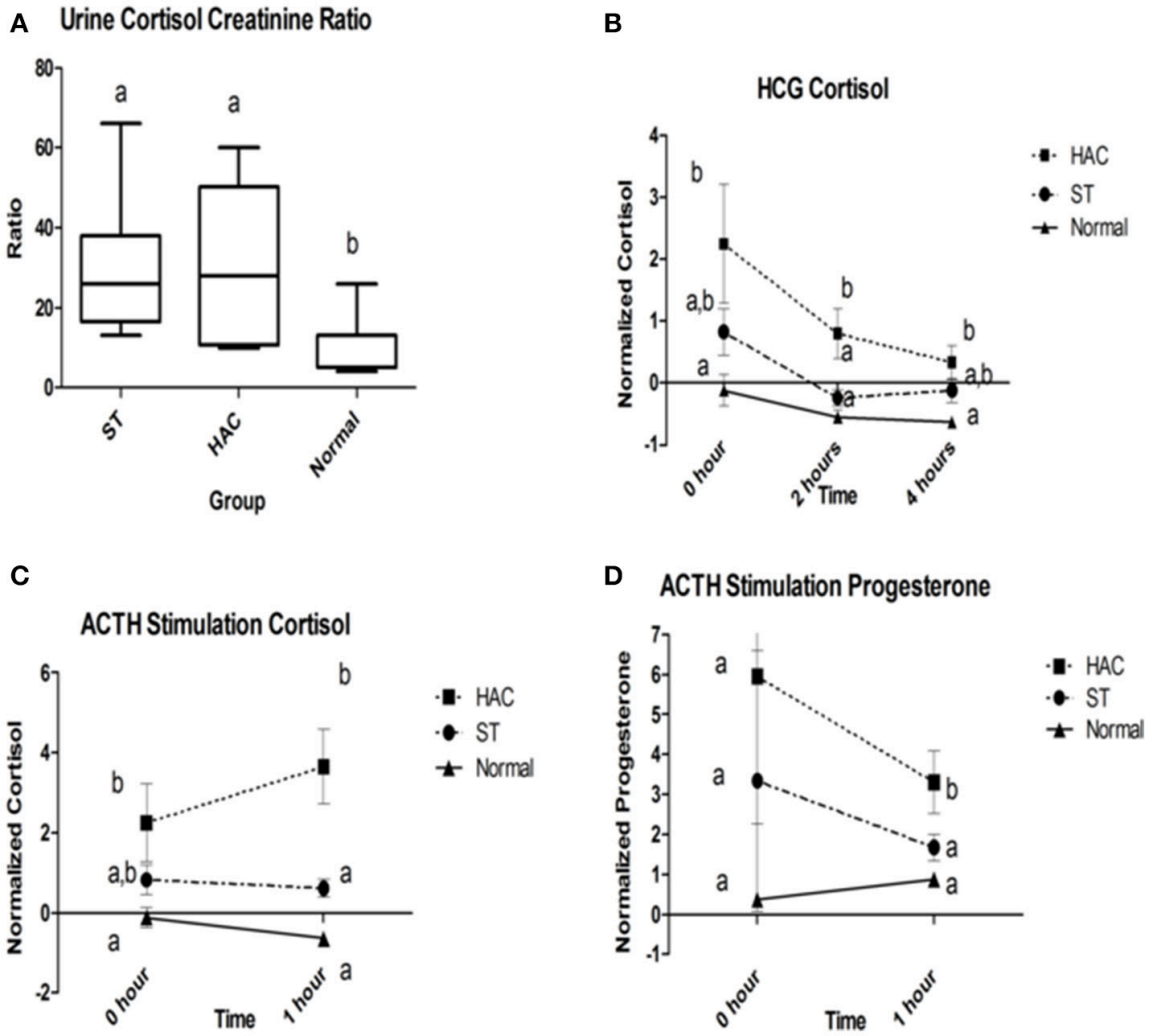
**(A)** Group comparison of urine cortisol to creatinine ratios (UCCR); **(B)** Normalized serum cortisol at 2 and 4 h following human chorionic gonadotropin (HCG) challenge; **(C,D)** Normalized serum cortisol and progesterone baseline and 1 h following adrenocorticotropic hormone (ACTH) administration. Data points with differing lowercase letter superscripts at same time point were significantly different (*p* < 0.05); error bars on **(B–D)** represents SEM. Group: ST, Scottish terriers with increased ALP activity (>210 U/L); HAC, dogs of non-Scottish terrier breed with pituitary dependent hyperadrenocorticism; Normal, normal dogs of non-Scottish terrier breed.

**Figure 3:**
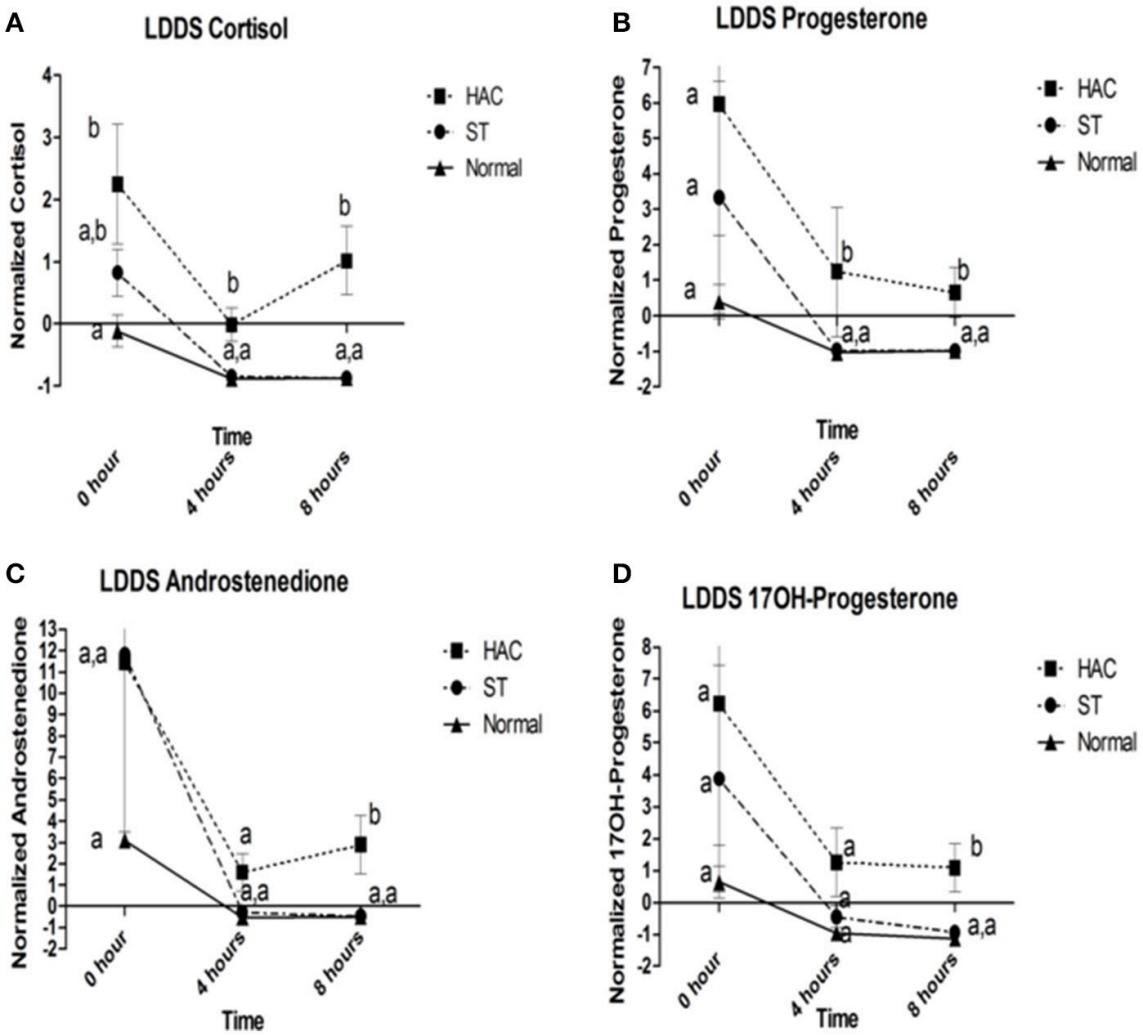
**(A–D)** Cortisol, progesterone, androstenedione, and 17OH-progesterone baseline, 4 and 8 h following low dose dexamethasone suppression (LDDS) challenge. Data points with differing lowercase letter superscripts at same time point were significantly different (*p* < 0.05); error bars represents SEM. Group: ST, Scottish terriers with increased ALP activity (>210 U/L); HAC, dogs of non-Scottish terrier breed with pituitary dependent hyperadrenocorticism; Normal, normal dogs of non-Scottish terrier breed.

Reference intervals for adrenal hormones are shown in Table 1. All HAC dogs (6/6) had a post-ACTH cortisol concentration above the reference interval, while it was elevated in only 2 of 8 ST and none of the Normal dogs, Table 2. Post-ACTH concentrations of one or more of the sex steroids was elevated in all ST and HAC dogs, while they were elevated in only 2 of the 7 Normal dogs. Hormone concentrations with mean group differences are shown in Table 3, Figures 2, 3. Urine cortisol/creatinine ratio was higher in the ST and HAC groups than in the Normal group, but there was no difference between the ST and HAC groups. The mean post-ACTH cortisol and progesterone concentrations were higher in the HAC group than in the ST and Normal groups, but no differences were found between ST and Normal dogs. Basal and 8 h post-dexamethasone serum concentrations of cortisol, progesterone, 17-OH progesterone, and androstenedione were higher in the HAC group compared with the ST and Normal groups, but no difference was found between the ST and Normal groups. Similarly, no differences were found between the adrenal steroid profile of ST and normal dogs following dexamethasone at 4 and 8 h while HAC dogs had higher values, Table 3, Figure 3. Resting baseline cortisol prior to HCG administration did not differ from initial testing 2 weeks prior (data not shown). The mean serum cortisol concentration before and 2 h after HCG was higher in the HAC group than the ST and Normal groups and was also higher in the HAC group than the Normal group at 4 h. No other significant differences in cortisol or sex steroid hormone concentrations were noted at any time between any groups.

**Table 1:** Reference intervals.

**Analyte**	**Sex**	**Baseline**	**LDDS^a^ 4, 8 h**	**Post-ACTH^b^**
Cortisol ng/mL	MN^c^ FS^d^	2.0–56.5 2.1–58.8	< 10 < 10	70.6–151.2 65.0–174.6
Androstenedione ng/mL	MN FS	0.05–0.36 0.05–0.57	NA NA	0.24–2.90 0.27–3.97
Estradiol pg/mL	MN FS	23.1–65.1 30.8–69.9	NA NA	23.3–69.4 27.9–69.2
Progesterone ng/mL	MN FS	0.03–0.17 0.03–0.49	NA NA	0.22–1.45 0.10–2.63
17 OH Progesterone ng/mL	MN FS	0.08–0.22 0.08–0.77	NA NA	0.25–2.63 0.40–1.62
Aldosterone pg/mL	MN/FS	11–139.9	NA	27.9–398.5
Adrenocorticotropic hormone	MN/FS	11.8–47.7	NA	NA
Urine Cortisol/Creatinine	M^e^/FS	8–24	NA	NA

*Adrenal steroid hormone reference intervals are from Clinical Endocrinology Service at the University of Tennessee; adrenocorticotrophic hormone interval is from Virginia Tech Animal Laboratory Services at Virginia Tech and urine creatinine cortisol ratio intervals are from Michigan State University Diagnostic Center for Population and Animal Health*.

a *Low dose dexamethasone suppression test*.

b *Adrenocorticotropic hormone stimulation test*.

c *Male neutered*.

d *Female spayed*.

e *Male*.

*NA, not applicable*.

**Table 2:** Tally of dogs with low, normal, and high adrenal hormones before and following combined low dose dexamethasone suppression (LDDS) and adrenocorticotropic (ACTH) stimulation tests.

**Analyte**	**Group**	**Number**	**Baseline**			**LDDS**^**a**^ **8 h**			**ACTH**^**b**^ **stim 1 h**		
			**Low**	**Normal**	**High**	**Low**	**Normal**	**High**	**Low**	**Normal**	**High**
Cortisol^c^	ST	8	0	5	3	0	8	0	0	6	2
	HAC	6	0	3	3	0	0	6	0	0	6
	Normal	7	0	7	0	0	6	1	1	7	0
Androstenedione^d^	ST	8	0	0	8	0	8	0	0	0	8
	HAC	6	0	1	5	0	0	4	0	1	5
	Normal	7	0	4	3	0	7	0	0	6	1
Estradiol^d^	ST HAC Normal	8 6 7	0 0 0	1 0 5	7 6 2	0 0 0	1 0 4	7 6 3	0 0 0	3 0 5	5 6 2
Progesterone^d^	ST HAC Normal	8 6 7	0 0 0	6 2 5	2 4 2	6 0 6	2 4 1	0 2 0	0 0 0	1 0 5	7 6 2
17 OH Progesterone^d^	ST HAC Normal	8 6 7	0 0 0	5 3 4	3 3 2	4 0 5	4 3 2	0 3 0	0 0 0	3 3 5	5 3 2
Aldosterone^d^	ST HAC Normal	8 6 7	0 0 1	6 5 6	2 1 0	0 0 1	8 4 6	0 2 0	0 0 0	4 3 7	4 3 0

*Reference values used for determination of Low, Normal, High shown in Table 1. Group: ST, Scottish terriers with increased ALP activity (>210 U/L); HAC, dogs of non-Scottish terrier breed with pituitary dependent hyperadrenocorticism; Normal, normal dogs of non-Scottish terrier breed*.

a *Low dose dexamethasone suppression test*.

b *Adrenocorticotropic hormone*.

c *Low dose dexamethasone suppression test cortisol < 10 ng/mL considered normal*.

d *Low dose dexamethasone suppression test: Low, normal, and high determination made using baseline reference intervals*.

**Table 3:** Adrenal related hormones with significant mixed-mode ANOVA response differences (*p* < 0.05, differing superscripts) resting and following combined low dose dexamethasone suppression (LDDS) and adrenocorticotropic hormone (ACTH), and human chorionic gonadotropin (HCG) challenge tests.

**Challenge**	**Time hours**	**Variable**	**Units^a^^,^^b^**	**ST**			**HAC**			**Normal**			***P*-value^c^**
				**Mean**	**Median**	***SD***	**Mean**	**Median**	***SD***	**Mean**	**Median**	***SD***	
Baseline	0	Cortisol	Normalized	0.825^AB^	0.45	1.074	2.250^A^	1.4	2.366	−0.114^B^	−0.4	0.665	0.015
			ng/mL	53.3	44.0	29.0	92.2	71.0	63.1	26.7	18.4	19.0	
		Urine Cortisol/Creatinine	None	30^A^	26	17	31^A^	28	20	9^B^	5	8	0.033
ACTH^d^	1	Cortisol	Normalized	0.638^A^	0.431	0.595	3.662^B^	3.527	2.285	−0.623^A^	−0.553	0.472	0.001
			ng/mL	147.6	143.4	28.9	283.5	270.1	99.2	86.6	89.5	19.5	
		Progesterone	Normalized	1.687^A^	1.65	0.954	3.307^B^	2.763	1.896	0.865^A^	0.7	0.498	0.043
			ng/mL	1.93	1.94	0.62	3.00	2.62	1.33	1.39	1.29	0.34	
LDDS^e^^,^^f^	4	Cortisol	Normalized	−0.857^A^	−0.8377	0.056	−0.023^B^	−0.224	0.665	−0.876^A^	−0.9266	0.101	0.001
			ng/mL	6.0	6.6	1.8	29.0	24.0	17.9	5.4	4.0	2.8	
		Progesterone	Normalized	−0.998^A^	−1.0217	0.124	1.220^B^	−0.57	4.45	−1.063^A^	−1.1429	0.109	0.004
			ng/mL	0.03	0.03	0.01	0.21	0.09	0.30	0.03	0.02	0.01	
	8	Cortisol	Normalized	−0.876^A^	−0.8845	0.069	1.021^B^	0.476	1.346	−0.866^A^	−0.9436	0.176	0
			ng/mL	5.5	5.2	2.0	58.3	43.2	38.0	5.7	3.6	5.0	
		Progesterone	Normalized	−1.016^A^	−1.0435	0.136	0.666^B^	−0.211	1.711	−1.020^A^	−1.1429	0.261	0.004
			ng/mL	0.03	0.02	0.01	0.27	0.12	0.33	0.04	0.02	0.05	
		Androstenedione	Normalized	−0.478^A^	−0.615	0.304	2.880^B^	1.49	3.34	−0.499^A^	−0.615	0.618	0.004
			ng/mL	0.17	0.15	0.08	0.82	0.59	0.57	0.16	0.13	0.16	
		17-OH progesterone	Normalized	−0.932^A^	−1	0.412	1.089^B^	1	1.862	−1.138^A^	−1.116	0.616	0.013
			ng/mL	0.08	0.08	0.03	0.39	0.22	0.39	0.09	0.07	0.09	
HCG^g^	2	Cortisol	Normalized	−0.248^A^	−0.363	0.371	0.810^B^	0.317	1.004	−0.532^A^	−0.661	0.281	0.013
			ng/mL	22.9	20.1	9.9	52.2	38.6	27.3	15.1	11.6	8.0	
	4	Cortisol	Normalized	−0.121^AB^	−0.317	0.525	0.316*A*	0.179	0.642	−0.616*B*	−0.6587	0.217	0.048
			ng/mL	26.5	21.0	14.6	38.4	35.3	17.3	12.7	11.3	6.2	

*Normalized values in gray used for statistical analyses. Means with differing superscript letters are statistically different. ST, Scottish terriers with increased ALP activity (>210 U/L); HAC, dogs of non-Scottish terrier breed with pituitary dependent hyperadrenocorticism; Normal, normal dogs of non-Scottish terrier breed*.

a Normalized values no units and used for statistical analyses; ACTH, LDDS and HCG results normalized using Tennessee Endocrinology Laboratory sex group baseline reference interval for each hormone such that normalized baseline reference interval equals “−1” to “+1.”

b *ACTH, LDDS, HCG normalized result = (actual result – [{lower reference interval + upper reference interval}/2])/([upper reference interval – lower reference interval value]/2)*.

c *Benjamini–Hochberg method*.

d *Adrenocorticotropic hormone stimulation test*.

e *Low dose dexamethasone suppression test*.

f *Log transformed*.

g *Human chorionic gonadotrophic challenge test*.

#### Pituitary

**Figure 4:**
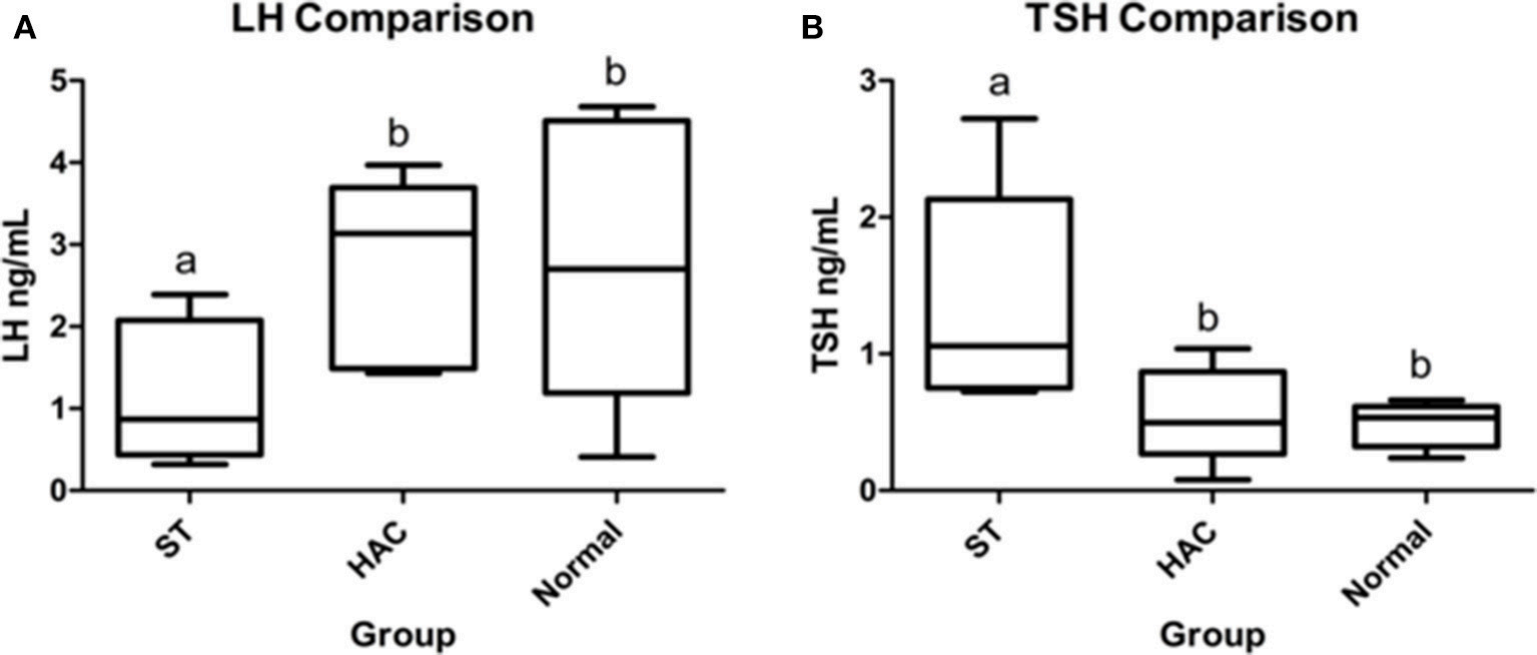
**(A,B)** Resting serum luteinizing hormone (LH) and thyroid stimulating hormone (TSH) group comparisons. Groups with differing lowercase letter superscripts are significantly different (*p* < 0.05), bar represents median value. Group: ST, Scottish terriers with increased ALP activity (>210 IU/L); HAC, dogs of non-Scottish terrier breed with pituitary dependent hyperadrenocorticism; Normal, normal dogs of non-Scottish terrier breed.

No differences were detected between groups for resting endogenous ACTH. There were significant differences between groups for mean serum LH and TSH concentrations, with LH being lower in ST while TSH was higher compared with the other groups (Table 4, Figure 4).

**Table 4:** Descriptive statistics and one-way ANOVA response differences (*p* < 0.05, differing superscripts) for plasma adrenocorticotropic hormone (ACTH) and serum luteinizing hormone (LH), prolactin (PL), growth hormone (GH), and thyroid stimulating hormone (TSH).

**Assay**	**ST**			**HAC**			**Normal**			***P*-value^a^**
	**Mean**	***SD***	**Median**	**Mean**	***SD***	**Median**	**Mean**	***SD***	**Median**	
ACTH^b^ pg/mL	36.50^A^	41.30	26.60	40.5^A^	46.20	22.40	32.10^A^	28.80	20.00	0.928
LH^c^ ng/mL	**1.18**^A^	0.82	0.87	**2.80**^B^	1.11	3.14	**2.91**^B^	1.72	2.70	0.028
PL^d^ ng/mL	18.18^A^	17.56	12.87	11.40^A^	3.33	11.23	15.15^A^	7.50	12.20	0.592
GH^e^ ng/mL	2.11^A^	0.49	2.07	1.58^A^	0.21	1.57	2.29^A^	1.09	1.99	0.202
TSH^f^ ng/mL	**1.36**^A^	0.76	1.06	**0.54**^B^	0.34	0.50	**0.49**^B^	0.16	0.54	0.011

*Analytes with significantly different means bolded and labeled with differing superscript letters. Group: ST, Scottish terriers with increased ALP activity (>210 IU/L); HAC, dogs of non-Scottish terrier breed with pituitary dependent hyperadrenocorticism; Normal, normal dogs of non-Scottish terrier breed*.

a *Benjamini-Hochberg method*.

b *Adrenocorticotropic hormone*.

c *Luteinizing hormone*.

d *Prolactin hormone*.

e *Growth hormone*.

f *Thyroid stimulating hormone*.

### Part 2: genomic

#### RNA

The same 21 dogs and cohorts used for the Part 1 study were used in the mRNA analysis. Three hundred ninety-one genes were differentially expressed between groups HAC and Normal, 182 genes between ST and Normal and 125 genes between ST and HAC dogs, when using a False Discovery Rate (FDR) threshold of 0.05 or 5%. At the more stringent FDR threshold of 0.001 or 0.1%, 33 genes were differentially expressed between groups HAC and Normal, 13 between ST and Normal and 15 between ST and HAC dogs. Focusing on candidate genes with a pituitary adrenal axis functional association and steroidogenesis pathway, only three genes were differentially expressed between HAC and Normal dogs: (1) TRHDE (ENSCAFG00000000473, 10:13746701-14118059:1, thyrotropin-releasing hormone degrading enzyme), (2) GHITM (ENSCAFG00000015891, 4:32401616-32417208:1, growth hormone inducible transmembrane protein), and (3) GH1 (ENSCAFG00000012681, 9:11832265-11834123:1, Canis lupus familiaris growth hormone). No unique subset of genes was found in ST with significant expression difference from HAC and Normal dogs. Specifically, no difference in mRNA expression of the 21-hydroxylase gene (CYP21, CYP21A, ENSCAFG00000000712) was seen in ST vs. HAC and Normal.

#### SNP and GWAS

**Figure 5:**
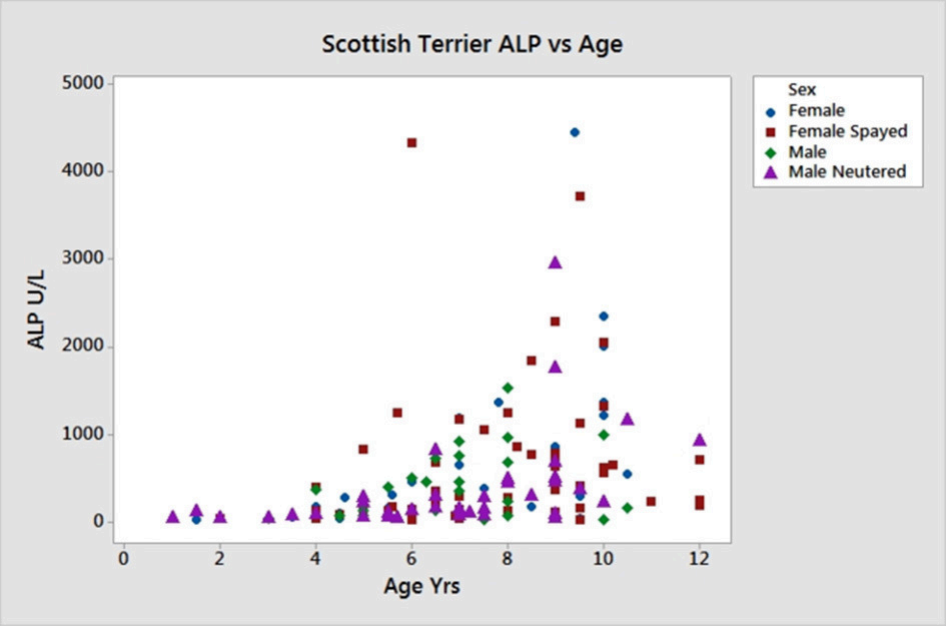
Age vs. serum ALP activity in 168 Scottish terriers showing relationship of increasing age and ALP activity.

Sex, age and ALP information on the 168 new ST enrolled for the SNP analysis are shown in Table 5. The linear effect of age on ALP was significant, Figure 5. Gender did not significantly affect ALP. Dogs with ALP >150 (>2 × upper limit of reference interval) were considered as “cases” (*n* = 95) and the remaining dogs as “controls” (*n* = 73). The logarithm base 2 of ALP (log2ALP) was approximately normally distributed. Age was significantly associated with log2ALP overall (*r* = 0.48, *p* = 4e-11), not significantly associated with log2ALP in controls (*r* = 0.14, *p* = 0.23), and significantly associated in cases (*r* = 0.30, *p* = 0.003). Age ranged from 1 to 12 years. ALP ranged from 23 to 4443, with an interquartile range from 74 to 656, and a median of 176.

**Table 5:** Descriptive statistics on the 168 Scottish terriers included in the genome-wide SNP study.

**Sex**	**Number**	**Age years**			**ALP**^**a**^ **U/L**		
		**Mean**	**Median**	**Range**	**Mean**	**Median**	**Range**
Female	37	6.8	7.0	1.5–10.5	555	170	30–4,443
Female spayed	62	7.3	7.0	2–12	568	214	23–4,326
Male	28	6.9	7.0	3–10.5	407	352	28–1,532
Male neutered	41	6.8	7.1	1–12	348	142	142–2,947

a *Alkaline phosphatase*.

Following quality control described in the materials and methods, a total of 83,447 SNPs remained for analysis. One dog with low call rate was excluded. A total of 156 (586, 2835) SNPs had *p*-values for Hardy-Weinberg equilibrium below 0.0001 (0.001, 0.01), which were flagged but not excluded (numbers were very similar between cases and controls). The median genomic kinship estimate was −0.005 with an IQR of −0.02 to 0.01, but for 54 pairs of dogs (0.3% of all pairs) the kinship estimate was at least 0.1, and for 4 pairs it was at least 0.2 (maximum 0.259).

**Figure 6:**
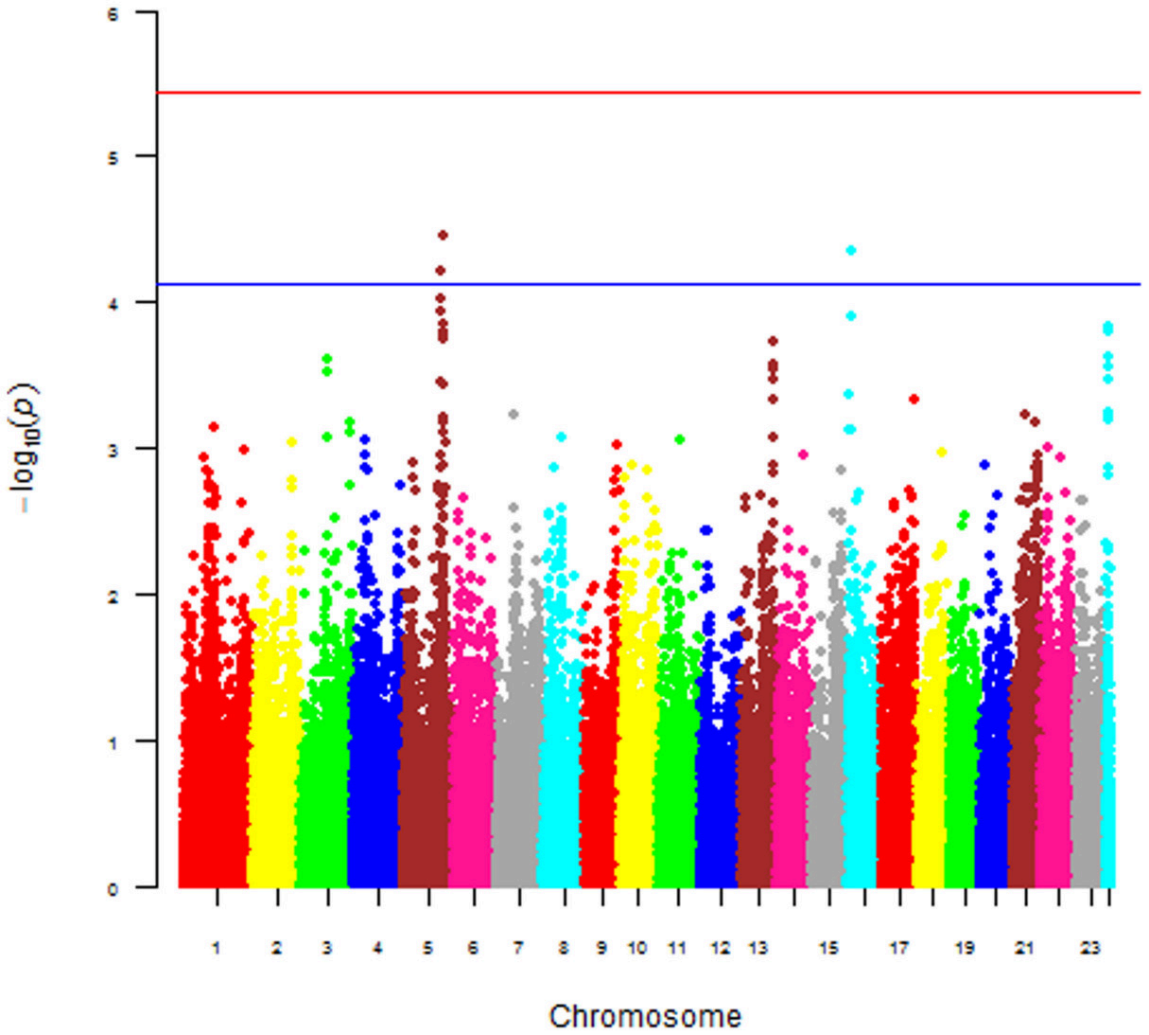
Manhattan of *p*-value against the genomic position of each SNP for the 168 Scottish terriers.

The main phenotype used for the GWAS was the case-control status of the dogs. The Manhattan plot shown in Figure 6 was obtained with this phenotype and plots the *p*-value [more precisely minus the logarithm base 10 of the *p*-value or –log10(p)] against the genomic position of each SNP. This plot revealed three areas (peaks) where p-values were smaller [hence –log10(p) values were larger] than the values everywhere else in the genome. The smallest *p*-values for the three peaks were 2.979e-05 (chromosome 5), 4.638e-05 (chromosome 16), and 0.000144 (chromosome 24), hence the *p*-values of all three peaks were larger than the stringent genome-wide significance threshold, but the *p*-values of the peaks on chromosomes 5 and 16 were below the suggestive significance threshold. We also performed a GWAS using the continuous log2ALP phenotype, but this analysis also did not produce any *p*-values below the significance threshold.

**Figure 7:**
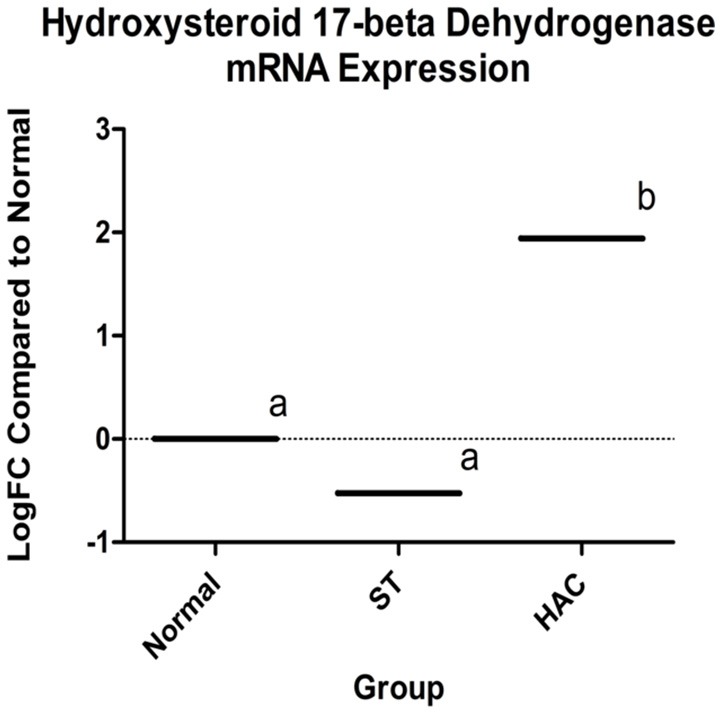
Log fold changes (logFC) between groups Normal, ST, and HAC mRNA expression levels of gene ENSCAFG00000019977, hydroxysteroid 17-beta with Normal dogs as reference; superscripts a, b indicates values with significant differences. Group: ST, Scottish terriers with increased ALP activity (>210 IU/L); HAC, dogs of non-Scottish terrier breed with pituitary dependent hyperadrenocorticism; Normal, normal dogs of non-Scottish terrier breed.

Known canine genes located within 1 mega base pairs from each of the three peak SNPs were collected and their functional annotations identified using DAVID (https://david.ncifcrf.gov). The suggestive peak area on chromosome 5 (with the smallest *p*-value) contained the gene HSD17B2, ENSCAFG00000019977 at a distance of 528 kilo base pairs from the SNP with the smallest *p*-value, which is involved in steroid hormone biosynthesis. Based on the GWAS identified area of interest on chromosome 5 and associated HSD17B2 gene, Part 1 mRNA data were revisited. Confirmatory analysis of the mRNA HSD17B2 data indicated that HAC dogs had significantly higher expression in comparison to ST (logFC 2.5) and Normal (logFC 1.9), Table 6, with expression differences greatest between HAC and ST dogs, Figure 7.

**Table 6:** Log fold changes (logFC) and *p*-values for pairwise comparisons between groups Normal, ST, and HAC mRNA expression levels of gene ENSCAFG00000019977, hydroxysteroid 17-beta dehydrogenase 2.

**Hydroxysteroid 17-beta dehydrogenase expression (gene ENSCAFG00000019977)**		
**Comparison**	**logFC**	***P*****-value**
HAC vs. Normal	1.942	0.024
ST vs. Normal	−0.526	0.526
HAC vs. ST	2.468	0.003

*Group: ST, Scottish terriers with increased ALP activity (>210 IU/L); HAC, dogs of non-Scottish terrier breed with pituitary dependent hyperadrenocorticism; Normal, normal dogs of non-Scottish terrier breed*.

## Discussion

The findings in this study support that there is excess adrenocortical hormone secretion in ST with elevated ALP, similar to our previous study (2). Concentrations of androstenedione were above reference intervals at baseline and after ACTH administration in all ST and in only one dog in the Normal group. Progesterone and 17-OH progesterone were also above respective reference intervals after ACTH in most ST. Further evidence of excess adrenocortical hormone secretion was provided by the significantly higher UCCR in ST compared with the Normal group.

Suppression of cortisol and other measured adrenocortical hormones was marked in the ST in response to dexamethasone and with a pattern similar to the control group. Given these findings, there was no indication of functional abnormalities between pituitary corticotrophs and the adrenal cortex in these ST. This is in marked contrast to dogs with HAC which had resistance to negative feedback on the LDDS test. Statistical analyses supporting these conclusions were strong. However, given the small group size, these interpretations should be viewed with caution until verified in a large study. Until that time, it does not appear that a corticotroph abnormality is responsible for the excess steroid hormone secretion in the ST.

Because of the apparent normal pituitary-adrenal axis in ST, it is possible that the pathogenesis of excess adrenocortical hormone secretion could be the result of aberrant receptors on adrenocortical cells. G-protein-coupled receptors responding to LH, glucose-dependent insulinotropic peptide (GIPR), serotonin, vasopressin, and glucagon have been identified in some humans with ACTH-independent Cushing's syndrome. mRNA for LH, GIPR, and vasopressin receptors has been identified in normal canine adrenal glands, with LH and vasopressin receptors being expressed in the cells (51–53). Receptors to all three of these hormones are expressed on canine adrenal tumor cells. It is possible that overexpression of eutopic or expression of other aberrant receptors could result in excess adrenal steroid secretion. Dogs in the present study were evaluated for an abnormal adrenocortical response to LH administration, but found no abnormalities in steroid hormone response to HCG in ST. Therefore, there is no evidence in this study that overexpression of LH receptors in adrenocortical cells is the cause of abnormal adrenal steroid secretion in these ST.

In contrast to GH and PL concentrations which were similar between ST and the control group, TSH was increased and LH was decreased in the ST. The significance of the higher TSH in ST is unclear, as serum thyroxine was not measured concurrently.

While a limitation of the clinical study was the small number of dogs in each group, the purpose was to explore the pathogenesis of the excess adrenocortical hormone secretion in ST identified in other studies (2, 3). Adrenocortical hormones in ST of the present study were very similar to those demonstrated previously (2, 3). This study adds additional information to the literature suggesting the regulatory pituitary adrenal axis of these ST was intact based on the consistent suppression of hormone secretion after dexamethasone administration. However, given the small group size, further studies are needed to investigate this conclusion and the lack of LH's role in the development of this disorder.

In the genome-wide mRNA profiling experiment, we did not find evidence that ST had decreased mRNA expression associated with the 21-hydroxylase gene as occurs most commonly in human cases of CAH. The SNP array data suggested there was a genomic region on canine chromosome, which contained a SNP associated with case-control status and ALP activity at the suggestive significance level, and which contains the HSD17B2 gene, an important enzyme in the cellular metabolism of estrogen, progesterone and testosterone. HSD17B2 expression differences were of interest for reasons of the GWAS SNP analysis, although these differences only reached point-wise but not genome-wide significance. HSD17B2 was over-expressed in the dogs with hyperadrenocorticism in comparison to both the normal and ST dogs; and, while not significant, it was slightly under-expressed in ST in comparison to normal dogs. This is surprising given the prevalence of increased sex steroids in this breed.

Given that HSD17B2 is responsible for the intracellular inactivation of sex steroids, particularly testosterone in the liver, it is intriguing to speculate that it plays a role in the pathogenesis of atypical hyperadrenocorticism in ST. In the HAC cohort, adrenal steroid biosynthesis and metabolism would be expected to be normal. Given the increased concentration of adrenal sex steroids in these dogs, increased expression of HSD17B2, involved in their degradation, was present as would be expected. Scottish terriers in this and prior studies have been shown to have increased concentrations of adrenal sex steroids (3). It would be reasonable to expect increased HSD17B2 mRNA expression in ST for the same reason it was increased in the hyperadrenocorticism cohort, i.e., homeostasis response to elevated adrenal sex steroids. The question then becomes, why is HSD17B2 mRNA expression not increased in ST? We acknowledge the mRNA data does not necessarily reflect end-gene product concentration but the expression differences between these groups is noteworthy and provides possible insight on the pathogenesis for increased sex steroids in this breed.

We have shown that a region on chromosome 5 which contains the HSD17B2 gene has a SNP associated with case-control status (ALP activity) of Scottish terriers. We speculate that this polymorphism could be linked with HSD17B2 gene silencing by some undetermined mechanism, e.g., microRNA, small interfering RNA, or long non-coding RNA. Since none of these genomic possibilities were directly assessed in this study, the nature of the potential association between the SNP polymorphism on chromosome 5 and increased ALP activity remains untested. However, if silencing or suppression of HSD17B2 gene is occurring in these ST, prolonged intracellular concentrations of sex steroids could be a contributing factor for hepatic glycogen accumulation and ALP changes as reported in this breed. Further studies will be required to validate these various findings and conclusions.

## Data availability

Raw data supporting the conclusions of this manuscript are available at Virginia Tech, VTechData open access data repository, DOI: 10.7294/5mx0-qs21, https://doi.org/10.7294/5mx0-qs21.

## Author contributions

KZ, DP, and WM: study design, data collection and analysis, manuscript preparation. IH: study design, data analysis, and manuscript preparation. MT, TL, and KF: sample processing and testing, manuscript preparation. SW: study design, statistical analysis, and manuscript preparation. BL: sample processing and manuscript preparation.

### Conflict of interest statement

The authors declare that the research was conducted in the absence of any commercial or financial relationships that could be construed as a potential conflict of interest.

## Abbreviations

ST: Scottish Terriers
ALP: alkaline phosphatase
LDDS: low dose dexamethasone test
HCG: human chorionic gonadotropin
ACTH: adrenocorticotrophic hormone
HAC: hyperadrenocorticism
SNP: single nucleotide polymorphisms
LH: luteinizing hormone
CAH: congenital adrenal hyperplasia
PD: polydipsia
PU: polyuria
UCCR: urine cortisol: creatinine ratio
TSH: thyroid stimulating hormone
PL: prolactin
GH: growth hormone
EDTA: ethylenediaminetetraacetic acid
GWAS: genome wide association study
BH: Benjamini-Hochberg
CPM: counts per million
TMM: trimmed mean of M-values
PKA: protein kinase A
GNAS: G protein alpha subunit gene.
